# Pterostilbeno Reduz o Estresse Oxidativo no Pulmão e no Ventrículo Direito Induzido por Infarto do Miocárdio Experimental

**DOI:** 10.36660/abc.20201155

**Published:** 2022-02-14

**Authors:** Silvio Tasca, Cristina Campos, Denise Lacerda, Vanessa D. Ortiz, Patrick Turck, Sara E. Bianchi, Alexandre L. de Castro, Adriane Belló-Klein, Valquiria Bassani, Alex Sander da Rosa Araújo

**Affiliations:** 1 Programa de Pós-Graduação em Ciências Biológicas: Fisiologia Instituto de Ciências Básicas da Saúde Universidade Federal do Rio Grande do Sul Porto Alegre RS Brasil Programa de Pós-Graduação em Ciências Biológicas: Fisiologia, Instituto de Ciências Básicas da Saúde, Universidade Federal do Rio Grande do Sul (UFRGS),Porto Alegre, RS - Brasil; 2 Programa de Pós-Graduação em Ciências Farmacêuticas Universidade Federal do Rio Grande do Sul Porto Alegre RS Brasil Programa de Pós-Graduação em Ciências Farmacêuticas, Universidade Federal do Rio Grande do Sul (UFRGS),Porto Alegre, RS - Brasil

**Keywords:** Antioxidantes, Estresse Oxidativo, Óxido Nítrico Sintase, Homeostase, Hormese, Pterostilbeno, NADPH Oxidases, Infarto do Miocárdio, Ratos

## Abstract

**Fundamento:**

O pterostilbeno (PS), um composto polifenólico natural e antioxidante, surge como uma intervenção promissora para minimizar danos do infarto agudo do miocárdio (IAM).

**Objetivo:**

Este estudo teve como objetivo avaliar o desempenho do PS na promoção da homeostase redox nos pulmões e no ventrículo direito (VD) de animais infartados.

**Métodos:**

Ratos Wistar machos (60 dias de idade) foram randomizados em três grupos: SHAM, IAM (infarto) e IAM+PS (IAM + pterostilbeno). Sete dias após o procedimento de IAM, os ratos foram tratados com PS (100 mg/kg/dia) por gavagem por oito dias. Os animais foram depois sacrificados e os pulmões e VD foram coletados para análise do balanço redox (diferenças foram consideradas significativas quando p<0,05).

**Resultados:**

Nossos resultados mostram que o IAM desencadeia a interrupção redox no VD e nos pulmões, o que pode contribuir para danos induzido pelo IAM nesses órgãos. Consistentemente, o PS mitigou o estresse oxidativo e restaurou as defesas antioxidantes (Glutationa – GSH nos pulmões: SHAM = 0,79 ± 0,07; IAM = 0,67 ± 0,05; IAM + PS = 0,86 ± 0,14; p<0,05), indicando seu papel protetor neste cenário.

**Conclusão:**

Nosso trabalho evidencia o potencial do uso de PS como abordagem terapêutica adjuvante após IAM para proteção dos tecidos pulmonares e cardíacos direitos.

## Introdução

O infarto agudo do miocárdio (IAM), evento agudo que ocorre quando o fluxo sanguíneo coronariano é interrompido, culmina em alterações hemodinâmicas, neuro-humorais e metabólicas que podem impactar negativamente a função pulmonar.^1 , 2^ A remodelação cardíaca adversa pós-IAM induz modificação da geometria ventricular e deslocamento dos folhetos da válvula mitral, o que prejudica seu processo de fechamento e causa modificações prejudiciais que afetam ambos os ventrículos. Na verdade, o IAM ventricular esquerdo com regurgitação mitral pode levar a alterações hemodinâmicas nos vasos pulmonares, refletindo em última instância no aumento da pressão arterial pulmonar. Todos esses distúrbios podem desencadear hipertensão pulmonar secundária à cardiopatia esquerda.^3^ Nesse cenário, o aumento da resistência vascular pulmonar (RVP) compromete a câmara cardíaca direita em decorrência da pós-carga elevada do ventrículo direito (VD), levando ao aumento da espessura da parede e diminuição da contratilidade. Essas alterações culminam em resposta adaptative ruim, caracterizada por dilatação, disfunção e falência do ventrículo direito.^4 , 5^

Importantes mediadores associados ao dano cardiopulmonar induzido pelo infarto são as espécies reativas de oxigênio (ROS), cujas principais fontes são as NADPH oxidases, a xantina oxidase e as mitocôndrias.^6 - 8^ Nesse sentido, o sistema enzimático antioxidante — constituído principalmente por superóxido dismutase (SOD), catalase (CAT) e glutationa peroxidase (GPx) — é o mecanismo central de defesa contra o dano celular induzido por ROS.^9^ Além do sistema enzimático antioxidante, os tecidos também podem recrutar antioxidantes não enzimáticos, como a glutationa reduzida.^10 , 11^ No entanto, foi relatado que a resposta antioxidante do VD é reduzida após o IAM.^12^ Nessa situação, a resposta contrarregulatória contra a ruptura da homeostase redox pode ser coordenada pelo fator de transcrição antioxidante, conhecido como fator nuclear eritroide 2 relacionado ao fator 2 (Nrf2).^13^ De fato, o Nrf2 regula a expressão de várias proteínas redox por meio da indução de elementos de resposta antioxidante (ERA), principalmente em condições de estresse oxidativo,^14^ e pode ser ativado por antioxidantes naturais, como o pterostilbeno (PS).^15^

O PS é um composto encontrado em uma grande variedade de frutas vermelhas, como mirtilos ( *Vaccinium spp.* ) E uvas ( *Vitis spp.* ). Quimicamente, corresponde ao resveratrol dimetilado (trans-3,5-dimetoxi-4’-hidroxiestilbeno), diferenciando-se dele por sua maior lipofilicidade.^15^ O mecanismo de ação dos estilbenos tem sido relacionado à redução dos níveis de ROS, como peróxido de hidrogênio e ânions superóxidos, bem como ao aumento da disponibilidade intracelular de antioxidantes enzimáticos e não enzimáticos.^16^ Nosso grupo relatou melhora nos parâmetros morfológicos do ventrículo esquerdo (VE) e nos marcadores de estresse oxidativo em ratos infartados tratados com PS (100 mg/kg/dia).^15^ No entanto, não existem estudos que avaliem se o composto pode causar uma atenuação dos danos induzidos pelo infarto nos pulmões e VD. Diante do exposto, o objetivo deste estudo foi avaliar o impacto do IAM sobre o estresse oxidativo no tecido pulmonar e no VD, e verificar se a administração de PS poderia melhorar a homeostase redox nesses órgãos.

## Métodos

### Química

O PS foi adquirido da Changsha Organic Herb (Changsha, China). A hidroxipropil--ciclodextrina (HPCD) foi fornecida pela Roquette Frères (Lestrem, França). A preparação e complexação do PS com HPCD para aumentar sua solubilidade em água foi conduzida como descrito previamente.^15^

### Ética

Ratos Wistar machos (60 dias de idade) foram obtidos no Centro de Reprodução e Experimentação de Animais de Laboratório da Universidade Federal do Rio Grande do Sul. Os animais foram alocados em caixas de polipropileno (340 x 200 x 410 mm) com três/quatro animais por gaiola. Foram mantidos em condições padrão: temperatura de 20-25° C, ciclos claro-escuro de 12 horas e umidade relativa de 70%. Água e rações comerciais foram oferecidas *ad libitum* . O protocolo experimental foi realizado de acordo com as Diretrizes Internacionais para Uso e Cuidado de Animais de Laboratório e do Conselho Nacional de Controle de Experimentação Animal. O protocolo só foi iniciado após aprovação pelo Comitê de Ética para Experimentação Animal da Universidade (#35451).

### Desenho experimental

Primeiramente, os animais foram divididos aleatoriamente em gaiolas e todas as avaliações foram realizadas às cegas.^15^ A cirurgia de infarto do miocárdio e a administração de PS foram realizadas de acordo com estudos anteriores do nosso grupo.^17^ A taxa de mortalidade durante o procedimento cirúrgico de infarto foi de 10%. Após a cirurgia, 17 ratos foram alocados nos seguintes grupos: SHAM (n = 6), grupo infartado (IAM) (n = 5) e grupo infartado tratado com PS (IAM + PS) (n = 6). A avaliação do tamanho da área de infarto do miocárdio foi realizada por meio de ecocardiografia (Philips HD7 XE Ultrasound System com transdutor L2-13 MHz), no dia 7 do protocolo experimental. Após essa avaliação, o PS foi administrado (100mg / kg / dia, por gavagem) para o grupo IAM + PS, e uma solução veículo (solução aquosa por gavagem) foi dada aos grupos SHAM e IAM por 8 dias. Após o período de tratamento, os animais foram sacrificados; os pulmões e os VDs foram coletados para análises morfométricas e bioquímicas.

### Avaliação ecocardiográfica

As análises ecocardiográficas (14 dias após o infarto) foram realizadas usando o sistema EnVisor Philips (Andover, MA, EUA) com um transdutor de alta frequência e alta resolução (12-3 MHz) por um operador treinado e com experiência em ecocardiografia de ratos, sem conhecimento do tratamento.^18^ Os animais foram anestesiados (cetamina 90 mg/kg; xilazina 20 mg/kg, i.p.) e colocados em decúbito lateral para obtenção das imagens. As imagens do ventrículo esquerdo (VE) foram avaliadas em três planos: basal, médio e apical. Os valores de encurtamento fracionário (EF) do VE foram obtidos pela seguinte equação: LVFS = DD – DS / DD × 100 (diâmetro diastólico – DD; diâmetro sistólico – DS). Os volumes sistólico e diastólico final do VE (VSF e VDF) foram medidos conforme descrito anteriormente.^18^ O débito sistólico (SO) foi calculado como SO = VDF – VSF.^19^ Em cada plano transversal ecocardiográfico (basal, médio e apical), o arco correspondente aos segmentos com infarto (regiões ou segmentos do miocárdio apresentando uma das seguintes alterações na cinética miocárdica: movimento sistólico acinesia e/ou região de hipocinesia – RHC) foi medido quanto ao perímetro infartado.^18 , 19^ A avaliação do perímetro infartado foi então usada para estimar o tamanho do infarto do miocárdio (R.B. Teixeira et al. Life Sciences 196 (2018) 93–101).

### Análise morfométrica dos ventrículos esquerdo e direito e pulmões

A eutanásia foi realizada por sobrecarga anestésica (cetamina 90 mg/kg e xilazina 10 mg/kg, por via intraperitonial) e confirmada por luxação cervical. Após a eutanásia, os pulmões, o VE e o VD foram utilizados para medidas morfométricas e bioquímicas. O pulmão esquerdo foi utilizado para determinar a razão pulmão/peso corporal, a fim de avaliar a congestão pulmonar. Para a realização dos índices de hipertrofia dos ventrículos direito e esquerdo, foram calculadas as razões ventrículo/peso corporal e ventrículo/comprimento da tíbia.^20^

### Preparação de homogenatos de pulmão e ventrículo direito

O pulmão direito foi preparado para as seguintes análises de estresse oxidativo: ROS total, peroxidação lipídica, glutationa total, glutationa reduzida, concentração de tiol, atividades de enzimas antioxidantes e expressão da proteína Nrf2. O VD foi homogeneizado para avaliar as atividades de NADPH oxidase e óxido nítrico-sintase, níveis de sulfidrila e imunoconteúdo de xantina oxidase. A homogeneização do pulmão e do VD foi realizada por 40 segundos com Ultra-Turrax (OMNI Tissue Homogenizer, OMNI International, EUA) na presença de KCl 1,15% (5 mL/g tecido) e fluoreto de fenilmetilsulfonil 100 mmol/L (PMSF). As amostras foram centrifugadas (20 minutos a 10.000 x g a 4° C), e o sobrenadante foi coletado e armazenado a 80° C até a análise.^21^ As análises bioquímicas foram realizadas por um pesquisador que desconhecia o tratamento.

### Avaliação de estresse oxidativo

No tecido pulmonar, a concentração total de ROS foi determinada pelo método de fluorescência por meio da reação com diacetato de dicloro fluoresceína (DCFH-DA) (Sigma-Aldrich, EUA). Os dados foram expressos em pmol/mg de proteína.^22^ A peroxidação lipídica foi medida pela reação dos produtos da oxidação com as substâncias reativas ao ácido tiobarbitúrico (TBARS) e os resultados apresentados em nmol/mg de proteína.^23^ Os níveis de glutationa total (GSH total) e dissulfeto de glutationa (GSSG) foram determinados pela redução de 5,5-ditiobis (ácido 2-nitrobenzóico) (DTNB) pela nicotinamida adenina dinucleotídeo fosfato (NADPH) catalisada pela glutationa redutase. Os dados são expressos como µmol/min/mg de tecido.^24^

### Atividade NADPH Oxidase

A atividade da enzima NADPH oxidase foi determinada pela avaliação do consumo de NADPH a 340 nm. Os resultados foram expressos em nanomoles de NADPH por minuto por miligrama de proteína (nmol/min/mg de proteína).^25^

### Determinação de antioxidantes enzimáticos e não enzimáticos

A atividade da SOD foi determinada por meio da inibição da auto-oxidação do pirogalol, e os resultados foram expressos em unidades de SOD/mg de proteína.^26^ A avaliação da atividade da CAT foi baseada no consumo de peróxido de hidrogênio, monitorando-se o decaimento da absorbância em 240 nm. Os resultados foram expressos em pmol/min/mg de proteína.^27^ A atividade da GPx foi estimada a partir da oxidação do NADPH, que foi acoplada à reação de reciclagem de GSSG para GSH, avaliada em 340 nm. Os resultados foram expressos em nmol/mg de proteína.^28^ A quantidade total de grupos sulfidrila no tecido pulmonar foi determinada pela reação dos grupos tiol com DTNB. A concentração de grupos sulfidrila totais foi expressa em nmol TNB/mg proteína.^29^ A concentração de proteína foi medida pelo método de Lowry.^30^

### Atividade de óxido nítrico-sintase

A atividade da enzima óxido nítrico-sintase foi avaliada medindo-se a conversão da oxihemoglobina (HbO2) em metemoglobina, induzida pelo óxido nítrico, conforme descrito anteriormente. Os valores são expressos em nmol NO/min/mg de proteína.^31^

### Avaliação do conteúdo imunológico de Nrf2 e xantina oxidase

O conteúdo imunológico de Nrf2 e xantina oxidase foram determinados por Western blot, conforme descrito anteriormente.^32^ Os anticorpos Nrf2 e a xantina oxidase foram usados como anticorpos primários (Santa Cruz Biotechnology, Santa Cruz, CA, EUA). Os anticorpos primários foram detectados por meio de anticorpos secundários conjugados com globulina de coelho anti-rato conjugada com peroxidase de rábano silvestre. As membranas foram desenvolvidas com reagentes de quimioluminescência. As autorradiografias foram escaneadas e as bandas foram medidas por meio de um software densitômetro (Imagemaster VDS CI, Amersham Biosciences Europe, IT). As bandas de pesos moleculares de Nrf2 e xantina oxidase foram determinadas por referência a um marcador de peso molecular padrão (RPN 800 rainbow full range Bio-Rad, CA, EUA). Os resultados foram normalizados pelo método de Ponceau.^33^

### Análise estatística

O cálculo do tamanho da amostra considerou uma probabilidade de erro = 0,05 e testou o poder estatístico (probabilidade de erro 1-) = 0,90. A distribuição dos dados foi avaliada pelo teste de Shapiro-Wilk. Como os dados apresentaram distribuição normal, os resultados foram analisados por ANOVA one-way com o teste post-hoc de Student-Newman-Keuls para detectar diferenças entre os grupos, e os resultados foram expressos em média ± desvio padrão (DP). As diferenças foram consideradas significativas quando p<0,05. Os dados foram analisados usando o software Sigma Plot (Jandel, Scientific Co, v. 11.0, San Jose, CA, EUA).

## Resultados

### Resultados morfométricos

Os ratos infartados de ambos os grupos (IAM e IAM + PS) tiveram um perímetro de infarto semelhante. Isso indica que não há diferenças entre os grupos infartados em termos de tamanho da lesão. A congestão pulmonar não diferiu entre os grupos experimentais. Da mesma forma, não houve alteração dos índices de hipertrofia do ventrículo direito, calculados pelas razões ventrículo direito/peso corporal e ventrículo direito/comprimento da tíbia, bem como do peso do ventrículo direito entre os grupos ( Tabela 1 ).

**Tabela 1:** Resultados morfométricos do pulmão e dos ventrículos esquerdo e direito

	SHAM (n=5)	IAM (n=5)	IAM+PS (n=6)
Pulmão/peso corporal (g/g)	4,01±0,99	5,99±0,44a	5,24±1,21
VD (g)	0,17±0,037	0,20±0,02	0,24±0,07
VD/comprimento da tíbia (g/cm)	0,48±0,09	0,57±0,07	0,66±0,19
VD/peso corporal (mg/g)	0,50±0,11	0,60±0,04	0,70±0,23
VE (g)	0,75±0,06	0,75±0,08	0,73±0,07
VE/comprimento da tíbia (g/cm)	2,05±0,19	2,09±0,22	2,00±0,18
VE/peso corporal (mg/g)	2,15±0,24	2,21±0,22	2,11±0,15

*Dados apresentados como média ± DP. ANOVA unilateral com o teste post-hoc de Student-Newman-Keuls. ap<0,05 vs SHAM. VD: ventrículo direito; VE: ventrículo esquerdo; SHAM: grupo controle; IAM: infarto do miocárdio; IAM + PS: infarto do miocárdio + pterostilbeno.*

### Parâmetros ecocardiográficos

Na avaliação dos parâmetros morfológicos do ventrículo esquerdo, tanto o grupo IAM quanto o IAM + PS apresentaram aumento dos volumes sistólico e diastólico final em comparação ao grupo SHAM, indicando dilatação ventricular. Porém, os animais IAM + PS apresentaram aumento do volume sistólico final mais discreto em relação ao IAM. O débito sistólico e a frequência cardíaca não foram diferentes entre os grupos. A fração de encurtamento do ventrículo esquerdo, que indica sua contratilidade, diminuiu nos grupos IAM e IAM + PS em relação aos animais SHAM, indicando piora na função sistólica desta câmara, e a administração de PS não foi eficaz para melhorar esse parâmetro. Em relação ao perímetro infartado, não houve diferença entre os grupos IAM e IAM + PS ( Tabela 2 ).

**Tabela 2:** Avaliação ecocardiográfica do ventrículo esquerdo

	SHAM (n=6)	IAM (n=5)	IAM+PS (n=6)
VSFVE (mL)	0,08±0,05	0,46±0,15 a	0,32±0,06 ab
VDFVE (mL)	0,29±0,09	0,68±0,14 a	0,57±0,06 a
Saída sistólica (mL)	0,21±0,04	0,22±0,03	0,24±0,04
Frequência cardíaca (bpm)	249±16	242±15	237±27
Fração de encurtamento (%)	51,5±5,4	15,8±1,7a	17,0±2,9a
Perímetro infartado (cm)	------------	1,81±0,45	1,60±0,22

*Dados apresentados como média ± DP. ANOVA unilateral com o teste post-hoc de Student-Newman-Keuls. ap <0,05 vs SHAM; bp <0,05 vs IAM. VSFVE: Volume sistólico final do ventrículo esquerdo; VDFVE: Volume diastólico final do ventrículo esquerdo; SHAM: Grupo controle; IAM: grupo infarto do miocárdio; IAM + PS: infarto do miocárdio + pterostilbeno. O perímetro infartado é um parâmetro ecocardiográfico de medição do tamanho do infarto.*

### Níveis de ROS, peroxidação lipídica e resposta antioxidante no tecido pulmonar

**Figura 1:**
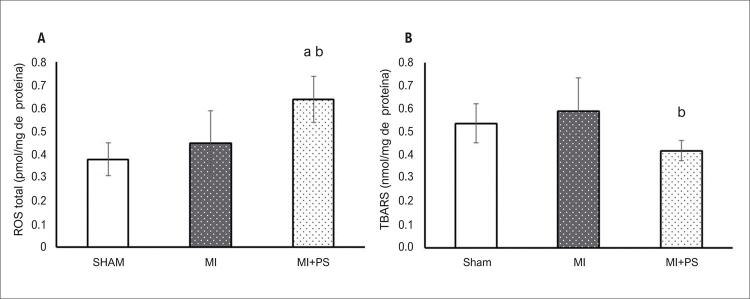
Estresse oxidativo pulmonar A) Concentração total de espécies reativas de oxigênio; B) Substâncias reativas ao ácido tiobarbitúrico. Dados expressos como média ± DP. ANOVA unilateral com o teste post-hoc de Student-Newman-Keuls. aP<0,05 vs SHAM; bP<0,05 vs IAM. SHAM: Grupo controle; IAM: grupo infarto do miocárdio; IAM+ PS: infarto do miocárdio + pterostilbeno.

O estresse oxidativo foi medido por meio da produção de dicloro fluoresceína (DCF) (um indicador dos níveis totais de ROS) e TBARS (um indicador de peroxidação lipídica) no tecido pulmonar. Em relação aos ROS totais, o grupo IAM + PS apresentou níveis aumentados em comparação aos grupos SHAM e IAM (p<0,05) ( Figura 1A ). No entanto, nenhuma diferença foi encontrada entre IAM e SHAM. Embora os níveis de espécies reativas tenham aumentado no grupo IAM + PS, a peroxidação lipídica diminuiu no tecido pulmonar desses animais em comparação com o grupo IAM (P<0,05). Além disso, a peroxidação lipídica no grupo IAM + PS não foi diferente em relação ao grupo SHAM, indicando redução do dano oxidativo promovida pela administração de PS ( Figura 1B ).

Em termos de defesas antioxidantes não enzimáticas, nenhuma mudança significativa nos níveis de GSH em ratos com IAM foi observada em comparação com ratos SHAM. Porém, o tratamento com PS teve efeito positivo nos pulmões dos animais IAM + PS, uma vez que os níveis de GSH aumentaram neste grupo quando comparados com SHAM e IAM (p<0,05) ( Tabela 3 ). No entanto, os níveis de GSSG, as relações GSH/Glutationa Total e GSSG/Glutationa Total não apresentaram diferenças entre os grupos.

**Tabela 3:** Parâmetros redox nos pulmões

	SHAM (n=6)	IAM (n=5)	IAM+PS (n=6)
GSH (µmol/min/mg de tecido)	0,79±0,07	0,67±0,05	0,86±0,14b
GSSG (µmol/min/ mg de tecido)	0,24±0,09	0,46±0,20	0,36±0,14
Glutationa total (µmol/min/mg de tecido)	1,27±0,13	1,40±0,22	1,55±0,33
GSH/ Glutationa total	0,63±0,11	0,49±0,12	0,57±0,15
GSSG/ Glutationa total	0,18±0,05	0,29±0,17	0,19±0,08

*Os dados são apresentados como média ± DP. ANOVA unilateral com o teste post-hoc de Student-Newman-Keuls bp<0,05 vs IAM. SAHM: Grupo controle; IAM: grupo infarto do miocárdio; IAM + OS: infarto do miocárdio + pterostilbeno; GSH: glutationa reduzida; GSSG: glutationa oxidada (unidade em µmol/min/mg de tecido).*

**Figura 2:**
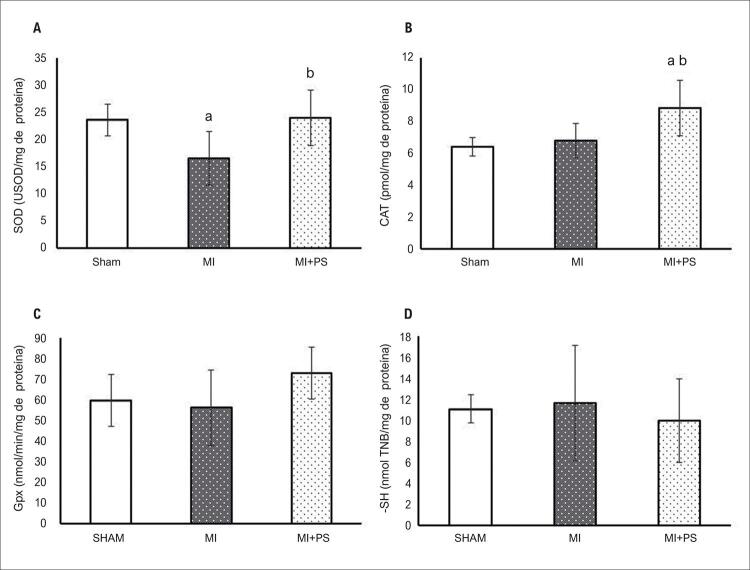
Medições de antioxidantes pulmonares. A) Atividade da superóxido dismutase; B) Atividade da catalase; C) Atividade da glutationa peroxidase; D) Grupos sulfidrila totais. Dados expressos como média ± DP. ANOVA unilateral com o teste post-hoc de Student-Newman-Keuls. aP<0,05 vs SHAM; bP<0,05 vs IAM. SHAM: Grupo controle; IAM: grupo infarto do miocárdio; IAM + PS: infarto do miocárdio + pterostilbeno .

Além da GSH, a administração de PS também parece melhorar as defesas antioxidantes enzimáticas nos pulmões. A atividade SOD foi reduzida no grupo IAM em comparação com o grupo SHAM. No entanto, essa atividade enzimática foi recuperada pelo PS (p<0,05) ( Figura 2A ). A CAT estava aumentada no grupo IAM + PS em relação aos grupos SHAM e IAM (p<0,05) ( Figura 2B ). A atividade da GPx e o SH total, entretanto, não se alteraram entre os grupos ( Figuras 2C e 2D , respectivamente).

### Expressão da proteína Nrf2 no tecido pulmonar

**Figura 3:**
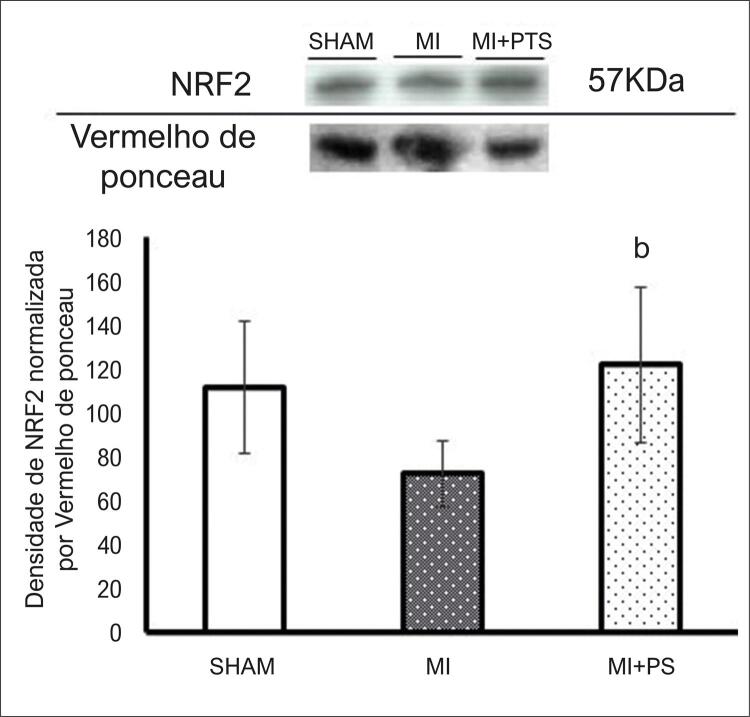
–Análise Western Blot da expressão de Nrf2 no pulmão. Gráfico representativo mostrando 1 banda para cada grupo experimental. Dados expressos como média ± DP. ANOVA unilateral com o teste post-hoc de Student-Newman-Keuls. bP <0,05 vs IAM. SHAM: Grupo controle; IAM: grupo infarto do miocárdio; IAM + PS: infarto do miocárdio + pterostilbeno .

A expressão de Nrf2 pode estar envolvida nos efeitos antioxidantes do PS. A Nrf2 é uma proteína relacionada com a regulação da transcrição de enzimas antioxidantes e pode ser estimulada por moléculas como os compostos fenólicos. De fato, nossos dados mostraram que o tratamento com PS promoveu um aumento significativo na expressão da proteína Nrf2 no grupo IAM + PS em comparação ao grupo IAM (p<0,05). No entanto, não houve diferença na expressão de Nrf2 entre os grupos SHAM e IAM ( Figura 3 ).

### Avaliação da Xantina Oxidase e NADPH Oxidase no VD

**Figura 4:**
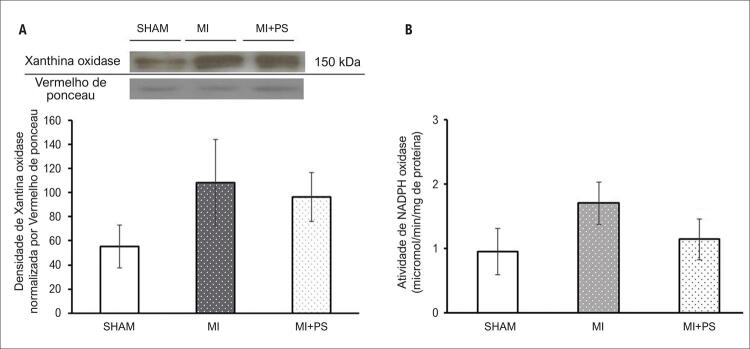
Estresse oxidativo do ventrículo direito. A) Análise Western Blot da expressão da xantina oxidase. Gráfico representativo mostrando 1 banda para cada grupo experimental; B) Atividade de NADPH oxidases. Dados expressos como média ± DP. ANOVA unilateral com o teste post-hoc de Student-Newman-Keuls. aP<0,05 vs SHAM; bP<0,05 vs IAM. SHAM: Grupo controle; IAM: grupo infarto do miocárdio; IAM + PS: infarto do miocárdio + pterostilbeno .

O infarto do miocárdio estimulou enzimas pró-oxidantes, que foram atenuadas pela administração de PS. Em relação a isso, a expressão da proteína xantina oxidase foi aumentada no VD de animais com IAM em relação aos demais grupos (p<0,05). No entanto, no grupo IAM + PS, os níveis de xantina oxidase não foram diferentes do grupo SHAM, o que indica uma atenuação dessa enzima pró-oxidante em animais infartados tratados com PS. Da mesma forma, houve um aumento na atividade da NADPH oxidase em animais infartados (IAM) em comparação com os do grupo SHAM, o que pareceu reduzido no grupo IAM + PS (p <0,05), comprovando a contribuição do tratamento com PS na redução da produção de radical ânion superóxido no VD ( Figura 4A e 4B ).

### Concentração de sulfidrila e atividade de NOS no VD

**Figura 5:**
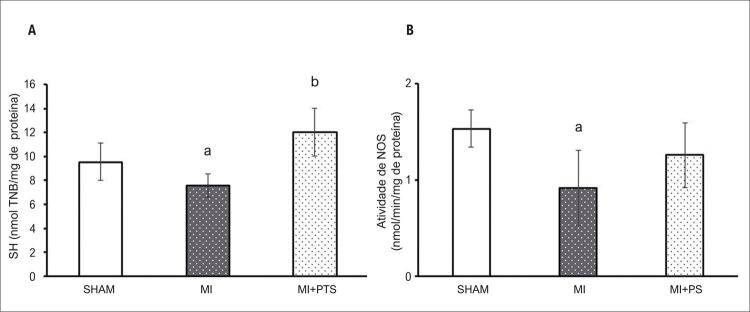
–A) Grupos sulfidrila total do ventrículo direito; B) Atividade da óxido nítrico-sintase do ventrículo direito. Dados expressos como média ± DP. ANOVA unilateral com o teste post-hoc de Student-Newman-Keuls. aP<0,05 vs SHAM; bP<0,05 vs IAM. SHAM: Grupo controle; IAM: grupo infarto do miocárdio; IAM + PS: infarto do miocárdio + pterostilbeno .

A concentração de sulfidrila (grupos tiol relevantes como antioxidantes não enzimáticos) foi diminuída no grupo IAM em comparação com o SHAM (p<0,05); entretanto, o grupo IAM + PS restabeleceu os níveis de sulfidrila (p<0,05). Além disso, ratos infartados não tratados apresentaram redução da atividade da NOS quando comparados aos animais SHAM, enquanto o tratamento com PS induziu a recuperação dessa atividade enzimática (p<0,05) ( Figura 5A e 5B ).

## Discussão

O principal achado deste estudo foi que o tratamento de ratos infartados com PS promoveu efeitos benéficos nos pulmões e no VD. Ao avaliar a morfologia e função do VE após o infarto, ambos os grupos infartados apresentaram dilatação cardíaca, refletida pelo aumento dos volumes cardíacos, e comprometimento da contratilidade, evidenciado pela diminuição da fração de encurtamento. A administração do PS, entretanto, atenuou o aumento do volume sistólico final, o que parece ser um resultado positivo, uma vez que o aumento do volume sistólico final pode estar relacionado ao desenvolvimento de congestão pulmonar. Na verdade, o tratamento com PS evitou a congestão pulmonar, conforme mostrado pela relação pulmão/peso corporal. O perímetro infartado não foi diferente entre os ratos IAM e IAM + PS, havendo homogeneidade de lesão cardíaca entre esses grupos. Em relação aos parâmetros morfométricos, nem o ventrículo direito nem o esquerdo dos grupos infartados apresentou hipertrofia. O fato de os animais terem sido avaliados apenas 14 dias após o infarto poderia explicar isso. Um estudo anterior ao nosso também não encontrou diferença nesses parâmetros.^32^

Os pulmões são os órgãos mais afetados pela insuficiência cardíaca, e a disfunção pulmonar é um fator-chave para desfechos clínicos ruins em pacientes infartados.^34^ No entanto, nosso estudo não encontrou alterações nos níveis totais de ROS nos pulmões de ratos infartados. Por outro lado, os pulmões de animais IAM + PS apresentaram níveis aumentados de ROS, o que está de acordo com o papel relatado dos estilbenos na indução da produção de ROS in vitro.^16^ O grupo IAM mostrou um aumento na peroxidação lipídica, indicando dano oxidativo nos pulmões. O grupo IAM + PS, entretanto, apresentou redução da lesão pulmonar induzida pela peroxidação lipídica, uma vez que os níveis de TBARS diminuíram. Uma possível hipótese é que esse aumento dos níveis de ROS ocasionado pela administração de PS poderia representar um mecanismo hormonal^35^ que leva ao aumento das defesas antioxidantes, evitando a peroxidação lipídica. De fato, estudos anteriores com outros compostos naturais que também apresentam efeito pró-oxidante, como o sulforafano, já reportaram esse mecanismo de proteção pela estimulação do sistema antioxidante.^36^ O tratamento com PS pode induzir uma adaptação contra o aumento dos níveis de ROS por meio de alterações oxidativas celulares na atividade de SOD e CAT, duas enzimas importantes que pertencem à primeira linha de defesa contra o estresse oxidativo^9^ Em nosso estudo, a redução da atividade da SOD nos pulmões do grupo IAM sugere proteção deficiente contra os radicais superóxido ânion, o que poderia causar um aumento do estresse oxidativo em estágios posteriores do IAM.^17^ Por outro lado, o tratamento com PS recuperou a atividade SOD no grupo IAM + PS, demonstrando seu efeito protetor na homeostase redox. Nossos resultados mostraram aumento da atividade CAT no tecido pulmonar de animais IAM + PS. Como o peróxido de hidrogênio pode reagir com metais como o ferro e produzir radicais hidroxila,^37^ esse aumento da atividade CAT no grupo IAM + PS surge como uma importante defesa contra a produção desse radical nos pulmões. Em termos de defesas não enzimáticas, foi encontrado um aumento na concentração de GSH nos pulmões de animais IAM + PS. GSH é o peptídeo antioxidante de baixo peso molecular mais prevalente^38^ e participa da regulação redox e da homeostase.^39^ Neste estudo, o aumento da concentração de GSH pode ter contribuído para a redução dos níveis de TBARS no grupo IAM + PS, diminuindo o estresse oxidativo nesses animais. Além da estimulação das defesas enzimáticas e não enzimáticas, o PS também foi capaz de induzir melhorias no perfil antioxidante dos tecidos pulmonares por meio da estimulação de proteínas citoprotetoras, como a Nrf2.^40^

Nesse contexto, a Nrf2 atua como um fator de transcrição sensível à redox, desempenhando um papel fundamental na resposta antioxidante pulmonar.40 Em situações de equilíbrio redox, a Nrf2 está ancorado na proteína 1, associada à ECH semelhante a Kelch (Keap1). Porém, quando há uma ruptura da homeostase redox, o complexo Nrf2-Keap1 se dissocia e libera Nrf2, que pode se translocar para o núcleo e iniciar a transcrição de moléculas antioxidantes.^41^ De fato, estudos têm demonstrado que o Nrf2 desempenha um papel importante na síntese de enzimas antioxidantes endógenas. Além disso, segundo Lacerda et al.^15^ o PS regula positivamente a expressão de Nrf2, o que aumenta o GSH celular e mitiga o dano oxidativo.^15^ Em nosso estudo, os níveis de imunoconteúdo Nrf2 estavam aumentados nos pulmões de ratos IAM + PS, sugerindo que o PS induz a ativação de Nrf2, o que poderia explicar a melhora nas defesas antioxidantes e redução da peroxidação lipídica. Portanto, o PS mostrou ser protetor para os pulmões após o infarto, pois evitou a congestão pulmonar, aumentou as atividades de SOD e CAT, aumentou os níveis de GSH e evitou a peroxidação lipídica, além de induzir Nrf2, uma proteína citoprotetora.

O cenário pró-oxidativo do infarto do miocárdio afeta significativamente o VD.^20^ Nossos resultados mostraram que o infarto do miocárdio leva à expressão elevada da xantina oxidase nele. Wang et al.^42^ mostraram aumento dos níveis de xantina oxidase no coração 12 semanas após o infarto, associado à peroxidação lipídica e disfunção cardíaca.^42^ Além disso, a atividade da NADPH oxidase, importante fonte de ROS, também foi avaliada em nosso estudo. A atividade da enzima foi elevada em ratos IAM, e a administração de PS foi capaz de prevenir esse aumento. Os níveis elevados de xantina oxidase e a atividade da NADPH oxidase predispõem o VD ao aumento da concentração do ânion superóxido e, consequentemente, à depleção das reservas antioxidantes. Corroborando esses resultados, também encontramos níveis reduzidos de sulfidrila no grupo IAM. O grupo IAM + PS, entretanto, apresentou conteúdo aumentado de grupos tiol. No grupo IAM, a concentração elevada de ânion superóxido produzida por NADPH e xantina oxidase pode interferir desfavoravelmente no equilíbrio de ROS/NO no VD. Na verdade, nossos resultados mostraram atividade reduzida de NOS na câmara direita de animais IAM. A NOS é uma enzima relevante na produção de NO, que desempenha papel fundamental na cardioproteção.^43^ No presente estudo, a administração de PS evitou a redução da atividade de NOS que ocorreu no grupo IAM. No entanto, considerando o efeito desse composto no embotamento de enzimas pró-oxidantes, como a NADPH oxidase e a xantina oxidase, concomitante à recuperação parcial da atividade da NOS, o PS parece contribuir para a manutenção de um equilíbrio cardioprotetor ROS/NO no VD.

## Conclusões

Em conclusão, a administração de PS promoveu efeitos benéficos nos pulmões de animais infartados, diminuindo a peroxidação lipídica e aumentando as defesas antioxidantes, como SOD, atividade de CAT e níveis de GSH. Também evitou o aumento da atividade da NADPH oxidase e da expressão da xantina oxidase no VD de animais infartados. Esses resultados provavelmente estão relacionados a uma melhora no equilíbrio ROS/NO nesta câmara. Portanto, nossos achados sugerem que o PS efetivamente tem efeitos protetores nos pulmões e no VD após IAM.

**Figura 6:**
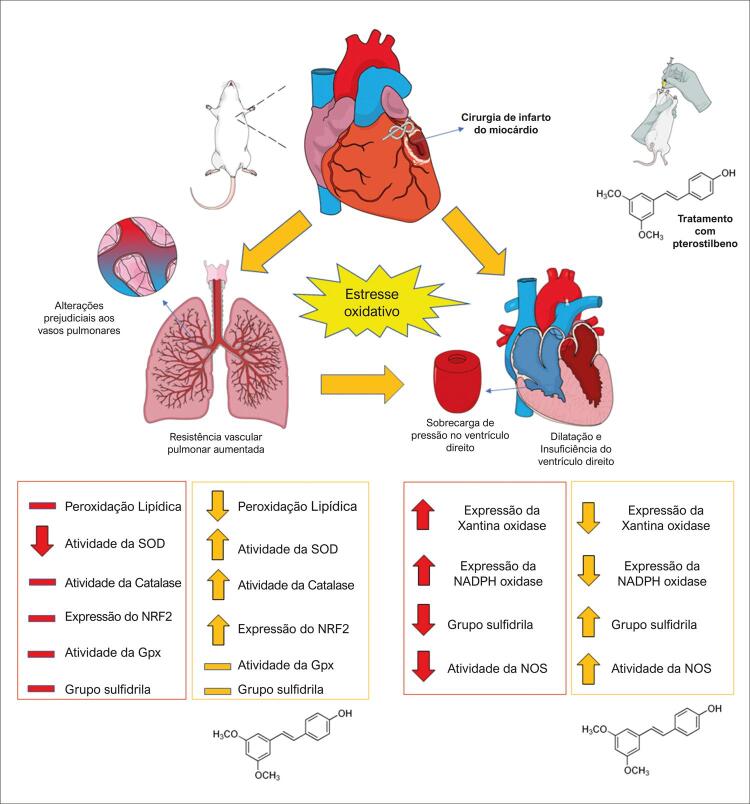
Resumo gráfico .
