# Supplementary material for: Pterostilbeno Reduz o Estresse Oxidativo no Pulmão e no Ventrículo Direito Induzido por Infarto do Miocárdio Experimental
Source: Arq Bras Cardiol. 2022 Feb 14;118(2):435–45. [Article in Portuguese] doi: 10.36660/abc.20201155 (PMC8856678; doi:10.36660/abc.20201155)
Supplement: Translation

# Translation: Pterostilbene Reduces Experimental Myocardial Infarction-Induced Oxidative Stress in Lung and Right Ventricle

**Authors:** Silvio Tasca, Cristina Campos, Denise Lacerda, Vanessa D. Ortiz, Patrick Turck, Sara E. Bianchi, Alexandre L. de Castro, Adriane Belló-Klein, Valquiria Bassani, Alex Sander da Rosa Araújo

**Affiliations:** 1 Brasil Programa de Pós-Graduação em Ciências Biológicas: Fisiologia, Instituto de Ciências Básicas da Saúde, Universidade Federal do Rio Grande do Sul (UFRGS), Porto Alegre, RS - Brasil; 2 Brazil Programa de Pós-Graduação em Ciências Farmacêuticas, Universidade Federal do Rio Grande do Sul (UFRGS), Porto Alegre, RS - Brazil

**Keywords:** Antioxidants, Oxidative Stress, Nitric Oxidase Synthase, Homeostasis, Hormesis, Pterostilbene, NADPH Oxidases, Myocardial Infarction, Rats

## Abstract

**Background:**

Pterostilbene (PS), a natural and antioxidant polyphenolic compound emerges as a promising intervention in improving the myocardial infarction (MI) damages.

**Objetives:**

This study aimed to evaluate PS actions in promoting redox homeostasis in lungs and right ventricle (RV) of infarcted animals.

**Methods:**

Male Wistar rats (60 day-old) were randomized into three groups: SHAM, MI (infarcted), and MI+PS (MI+pterostilbene). Seven days after MI procedure, rats were treated with PS (100 mg/kg/day) via gavage for eight days. Animals were euthanized and the lungs and RV were harvested for analyses of redox balance (Differences were considered significant when p<0.05).

**Results:**

Our results show that MI triggers a redox disruption scenario in RV and lungs, which can contribute to MI-induced damage on these organs. Consistently, PS mitigated oxidative stress and restored antioxidant defenses (GSH in lungs: SHAM= 0.79±0.07; MI=0.67±0.05; MI+PS=0.86±0.14; p<0.05), indicating its protective role in this scenario.

**Conclusions:**

Our work evidences the PS potential use as an adjuvant therapeutic approach after MI focusing on protecting pulmonary and right-sided heart tissues.

## Introduction

Myocardial infarction (MI), an acute event that occurs when coronary blood flow is interrupted, culminates in hemodynamic, neuro-humoral, and metabolic alterations, which can negatively impact pulmonary function.^1 , 2^ The adverse post-MI cardiac remodeling induces modification of ventricular geometry and shifting of mitral valve leaflets which impairs its closing process, causing detrimental modifications that affect both ventricles. In fact, left ventricular MI with mitral regurgitation can lead to hemodynamic changes in the pulmonary vessels, ultimately reflecting in increased pulmonary arterial pressure. All these disturbances can trigger pulmonary hypertension secondary to left heart disease.^3^ In this scenario, the increased pulmonary vascular resistance (PVR) compromises the right heart, as a result of elevated right ventricle (RV) afterload, leading to increased wall thickness and decreased contractility of this chamber. These changes culminate in poor adaptive response, characterized by right ventricular dilation, dysfunction and failure.^4 , 5^

Important mediators associated with the infarction-induced cardiopulmonary damage are the reactive oxygen species (ROS), whose main sources are NADPH oxidases, xanthine oxidase, and mitochondria.^6 - 8^ In this sense, the antioxidant enzymatic system, constituted mainly by superoxide dismutase (SOD),catalase (CAT), and glutathione peroxidase (GPx), represents the pivotal mechanism of defense against ROS-induced cellular damage.^9^ In addition to antioxidant enzymatic system, tissues can also recruit non-enzymatic antioxidants, such as reduced glutathione.^10 , 11^ However, antioxidant response of RV has been reported to be reduced after MI.^12^ In this situation, the counter regulatory response against redox homeostasis disruption may be coordinated by the antioxidant transcription factor known as nuclear factor 2 related to erythroid factor (Nrf2).^13^ Indeed, Nrf2 regulates the expression of several redox proteins through induction of the antioxidant response elements (ARE), mainly in oxidative stress conditions^14^ and can be activated by natural antioxidants, such as pterostilbene (PS).^15^

PS is a compound found in a wide variety of berries, such as blueberries ( *Vacciniumspp* ) and grapes ( *Vitisspp* ). Chemically it corresponds to dimethylated resveratrol (trans-3,5-dimethoxy-4’-hydroxy-stilbene), differing from it in terms of its higher lipophilicity.^15^ The mechanism of action of stilbenes has been related to the reduction of ROS levels, such as hydrogen peroxide and superoxide anions, as well as the increase in intracellular availability of enzymatic and non-enzymatic antioxidants.^16^ Our group has reported improvement in left ventricle morphological parameters, as well as in oxidative stress markers, in infarcted rats treated with PS (100 mg kg/day).^15^ However, there are no studies evaluating whether this compound could cause an attenuation of infarction-induced damage in the lungs and RV. In view of that, the objective of this study was to evaluate the impact of myocardial infarction on oxidative stress in the lung tissue and RV and explore whether PS administration could improve redox homeostasis in these organs.

## Methods

### Chemicals

PS was purchased from Changsha Organic Herb (Changsha, China). Hydroxypropyl--cyclodextrin (HPCD) was supplied by Roquette Frères (Lestrem, France). PS preparation and complexation with HPCDin order to enhance its water solubility was conducted as previously described.^15^

### Ethical

Wistar male rats (60 day-old) were obtained from the Center for Reproduction and Experimentation of Laboratory Animals of the Federal University of Rio Grande do Sul. The animals were allocated in polypropylene boxes (340 x 200 x 410 mm) with three/four animals per cage. Animals were kept under standard conditions: temperature (20–25°C), light–dark cycles of 12 hours and relative humidity of 70%. Water and commercial feed were offeredad *libitum* . The experimental protocol was carried out in accordance with the International Guidelines for Use and Care of Laboratory Animals and National Council for Control of Animal Experimentation. The protocol only started after the University’s Ethical Committee for Animal Experimentation had approved it (#35451).

### Experimental design

In the beginning of the experimental protocol, the animals were randomly divided and all assessments were performed blindly.^15^ Myocardial infarction surgery and PS administration were performed according to previous studies from our group.^17^ The rate of mortality during the infarction surgery procedure was 10%. After the surgery, 17 rats were allocated into the following groups: Sham (n=6), infarcted group (MI) (n=5), and infarcted group treated with PS (MI+PS) (n=6). The evaluation of myocardial infarction area size was performed using echocardiography (Philips HD7 XE Ultrasound System with an L2-13 MHz transducer), at the day 7 of the experimental protocol. After this evaluation, PS (100mg/kg/day, via gavage) for MI+PS group, and vehicle (aqueous solution via gavage) administrations for SHAM and MI groups, were started for 8 days. After the treatment period, animals were submitted to euthanasia; the lungs and right ventricles (RVs) were collected for morphometric and biochemistry analyses.

### Echocardiographic evaluation

The echocardiographic analyses (14 days after infarction) were performed using the EnVisor Philips system (Andover, MA, USA) with a high-frequency and high-resolution transducer (12-3 MHz), and conducted by a trained operator with experience in rat’s echocardiography and unaware of treatment.^18^ The animals were anesthetized (ketamine 90 mg/kg; xylazine 20 mg/kg, i.p.) and placed in lateral decubitus to obtain the images. Left ventricle (LV) images were assessed in three planes: basal, middle and apical. LV fractional shortening (FS) values were obtained by using the following equation: LVFS = DD − SD/DD × 100 (diastolic diameter — DD; systolic diameter — SD). LV end-systolic (ESV) and end-diastolic volumes (EDV) were measured as previously described.^18^ Systolic output (SO) was calculated as SO = EDV – ESV.^19^ On each echocardiographic transverse plane (basal, middle and apical) the arch corresponding to the segments with infarction (regions or segments of the myocardium showing one of the following changes in myocardial kinetics: systolic movement akinesis and/or hypokinesis region — AHR) were measured to indicate infarcted perimeter.^18 , 19^ The evaluation of infarcted perimeter was used to estimate myocardial infarction size.

### Morphometric analysis of left and right ventricles and lungs

Euthanasia was performed through anesthetic overload (ketamine 90 mg/kg and xylazine 10 mg/ kg, intraperitonially) and confirmed by cervical dislocation. After euthanasia, the lungs, left and right ventricle were used for morphometric and biochemical measurements. The left lung was used for the determination of the lung/body weight ratio, in order to evaluate lung congestion. In order to perform the hypertrophy indexes of the right and left ventricles, the ventricle/body weight and ventricle/tibia length ratios were calculated.^20^

### Preparation of lung and right ventricle homogenates

The right lung was prepared for the following oxidative stress analyses: total ROS, lipid peroxidation, total glutathione, reduced glutathione, thiol concentration, antioxidant enzyme activities, and Nrf2 protein expression. The RVwas homogenized to assay NADPH oxidases and nitric oxide synthase activities, sulfhydryl levels, and xanthine oxidase immunocontent. Lung and RV homogenization was performed for 40 seconds with Ultra-Turrax (OMNI Tissue Homogeneizer, OMNI International, USA) in the presence of 1.15% KCl (5 mL/g tissue) and 100 mmol/L phenyl methyl sulfonyl fluoride. Samples were centrifuged (20 minutes at 10000 x *g* at 4°C), and the supernatant was collected and stored at 80°C until the analyses.^21^ The biochemistry analyses were performed by researcher unaware of treatment.

### Oxidative stress evaluation

In pulmonary tissue, total ROS concentration was determined by the fluorescence method through reaction with dichlorofluorescein diacetate (DCFH-DA) (Sigma-Aldrich, USA). Data were expressed as pmol/mg protein.^22^ Lipid peroxidation was measured by the reaction of oxidation products with the thiobarbituric acid reactive substances (TBARS) and the results were represented as nmol/ mg protein.^23^ Total glutathione (GSH Total) and glutathione disulfide (GSSG) levels were determined by the reduction of 5,5-dithiobis (2-nitrobenzoic acid) (DTNB) by nicotinamide adenine dinucleotide phosphate (NADPH) catalyzed by glutathione reductase. Data were expressed as µmol/min/mg tissue.^24^

### NADPH Oxidase Activity

The activity of the NADPH oxidase enzyme was determined through the evaluation of NADPH consumption at 340 nm. The results were expressed as nanomoles of NADPH per minute per milligram of protein (nmol / min / mg protein).^25^

### Determination of enzymatic and non-enzymatic antioxidants

Superoxide dismutase (SOD) activity was determined through inhibition of pyrogallol auto-oxidation, and the results were expressed as units SOD/mg protein.^26^ Catalase (CAT) activity evaluation was based on the hydrogen peroxide consumption monitoring the absorbance decay at 240 nm Results were expressed as pmol/min/mg protein.^27^ Glutathione peroxidase (GPx) activity was estimated from the NADPH oxidation, which was coupled to recycling reaction from GSSG to GSH, evaluated at 340 nm. Results were expressed as nmol/mg protein.^28^ The total amount of sulfhydryl (SH) groups, in the lung tissue, was determined through thiol groups reaction with DTNB. The concentration of total sulfhydryl groups was expressed as nmol TNB/ mg protein.^29^ The protein concentration was measured by the method of Lowry.^30^

### Nitric oxide synthase enzyme activity

The activity of the nitric oxide synthase enzyme was evaluated by measuring the conversion of oxyhemoglobin (HbO2) to methemoglobin, induced by the presence of nitric oxide, as previously described. The values were expressed as nmol NO/min/mg protein.^31^

### Evaluation of Nrf2 and Xanthine oxidase immunocontent

The Nrf2 and xanthine oxidase immunocontents were determined by Western blot as previously described.^32^ Nrf2 and xanthine oxidase antibodies were used as primary antibodies (Santa Cruz Biotechnology, Santa Cruz, CA, USA). Primary antibodies were detected using anti-rabbit horseradish peroxidase-conjugate secondary antibodies. The membranes were developed using chemiluminescence reagents. The autoradiographs generated were scanned and bands were measured using a densitometer software (Imagemaster VDS CI, Amersham Biosciences Europe, IT). The Nrf2 and xanthine oxidase molecular weights bands were determined by reference to a standard molecular weight marker (RPN 800 rainbow full range Bio-Rad, CA, USA). The results were normalized by Ponceau red method.^33^

### Statistical analysis

The calculation of sample size were considered probability of error = 0.05 and test the statistical power (1- error probability) = 0.90. The distribution of data was evaluated by the Shapiro–Wilk test. Since the data presented normal distribution, the results were analyzed using one-way ANOVA with the Student-Newman-Keuls post-hoc test to detect differences between groups, and results were expressed as mean ± standard deviation (SD). Differences were considered significant when p<0.05. Data were analyzed using the Sigma Plot software (Jandel, Scientific Co, v. 11.0, San Jose, CA, USA).

## Results

### Morphometric results

The infarcted rats of both groups (MI and MI+PS) presented a similar infarction perimeter. This result indicates that there were no differences between the infarcted groups in terms of infarction size. Lung congestion had no difference among the experimental groups. In the same way, there was no change in the right ventricle hypertrophy indexes, calculated by right ventricle/body weight and right ventricle/tibia length ratios, as well as right ventricle weight among the groups ( Table 1 ).

**Table 1:** Morphometric results of lung and left and right ventricles

	SHAM (n=5)	MI (n=5)	MI+PS (n=6)
Lung/body weight (g/g)	4.01±0.99	5.99±0.44a	5.24±1.21
RV (g)	0.17±0.037	0.20±0.02	0.24±0.07
RV/tibia length (g/cm)	0.48±0.09	0.57±0.07	0.66±0.19
RV/body weight (mg/g)	0.50±0.11	0.60±0.04	0.70±0.23
LV (g)	0.75±0.06	0.75±0.08	0.73±0.07
LV/tibia length (g/cm)	2.05±0.19	2.09±0.22	2.00±0.18
LV/body weight (mg/g)	2.15±0.24	2.21±0.22	2.11±0.15

*Data are shown as mean ± SD. One-way ANOVA with the Student-Newman-Keuls post-hoc test. aP<0.05 vs SHAM. RV: Right ventricle; LV: Left ventricle; SHAM: Control group; MI: myocardial infarction group; MI + PS: myocardial infarction + pterostilbene.*

### Echocardiographic parameters

Evaluating morphologic parameters of the left ventricle, both MI and MI+PS groups presented an increase in end-systolic and end-diastolic volumes in comparison with SHAM animals, indicating ventricular dilatation. However, MI+PS animals presented a lower increase in end-systolic volume in relation to MI. Systolic output and heart rate were not different among the groups. Left ventricle fractional shortening, which indicates its contractility, was decreased in both MI and MI+PS groups in relation to SHAM animals, indicating a worsening in the systolic function of this chamber, and PS administration was not effective in improving this parameter. In relation to infarcted perimeter, there was no difference between MI and MI+PS groups ( Table 2 ).

**Table 2:** Echocardiographic evaluation of left ventricle

	SHAM (n=6)	MI (n=5)	MI+PS (n=6)
LVESV (mL)	0.08±0.05	0.46±0.15 a	0.32±0.06 ab
LVEDV (mL)	0.29±0.09	0.68±0.14 a	0.57±0.06 a
Systolic output (mL)	0.21±0.04	0.22±0.03	0.24±0.04
Heart rate (bpm)	249±16	242±15	237±27
Shortening Fraction (%)	51.5±5.4	15.8±1.7a	17.0±2.9a
Infarcted perimeter (cm)	------------	1.81±0.45	1.60±0.22

*Data are shown as mean ± SD. One-way ANOVA with the Student-Newman-Keuls post-hoc test. aP<0.05 vs SHAM; bP<0.05 vs MI. LVESV: Left ventricle end-systolic volume; LVEDV: Left ventricle end-diastolic volume; RV: Right ventricle; LV: Left ventricle; SHAM: Control group; MI: myocardial infarction group; MI + PS:*

### ROS levels, lipid peroxidation, and antioxidant response in lung tissue

Oxidative stress was measured via dichloroﬂuorescein (DCF) production (an indicator of total ROS levels) and TBARS (an indicator of lipid peroxidation) in the lung tissue. Regarding total ROS, MI+PS group showed increased levels as compared to both SHAM and MI (p<0.05) ( Figure 1A ). However, it was not observed any difference between MI and SHAM groups. Although reactive species levels were increased in MI+PS group, lipid peroxidation was decreased in the lung tissue of these animals compared to MI group (p<0.05). Besides that, lipid peroxidation in MI+PS group was not different compared to SHAM group, indicating a reduction of oxidative damage promoted by PS administration ( Figure 1B ).

**Figure 1:** Lung oxidative stress A) Total reactive oxygen species concentration; B) Thiobarbituric acid reactive substances. Data are expressed as mean ± SD. One-way ANOVA with the Student-Newman-Keuls post-hoc test. a P<0.05 vs SHAM; b P<0.05 vs MI. SHAM: Control group; MI: myocardial infarction group; MI + PS: myocardial infarction + pterostilbene.

In terms of non-enzymatic antioxidant defenses, there were no significant changes in GSH levels in MI rats compared to SHAM. However, in relation to this parameter, PS treatment demonstrated a positive effect in the lungs of MI+PS animals, since GSH levels were increased in this group when compared to SHAM and MI (p< 0.05) ( Table 3 ). Nevertheless, GSSG levels, GSH/Total glutathione and GSSG/Total glutathione ratios did not present differences among the groups.

**Table 3:** Redox parameters in the lungs

	SHAM (n=6)	MI (n=5)	MI+PS (n=6)
GSH (µmol/min/mg tissue)	0.79±0.07	0.67±0.05	0.86±0.14b
GSSG (µmol/min/ mg tissue)	0.24±0.09	0.46±0.20	0.36±0.14
Total glutathione (µmol/min/mg tissue)	1.27±0.13	1.40±0.22	1.55±0.33
GSH/ Total glutathione	0.63±0.11	0.49±0.12	0.57±0.15
GSSG/ Total glutathione	0.18±0.05	0.29±0.17	0.19±0.08


*Data are shown as mean ± SD. One-way ANOVA with the Student-Newman-Keuls post-hoc test b P<0.05 vs MI. Control group = SHAM; myocardial infarction group = MI, myocardial infarction + pterostilbene = MI+PS. GSH = reduced glutathione; GSSG = oxidized glutathione (unit in µmol/min/mg tissue).*

Besides GSH, PS administration also seems to improve the enzymatic antioxidant defenses in the lungs. SOD activity was reduced in MI group compared to SHAM. However, such enzymatic activity was recovered by PS (p<0.05) ( Figure 2A ). In addition, catalase presented an increased activity in MI+PS group in relation to both SHAM and MI groups (p<0.05) ( Figure 2B ). Both GPx activity and total –SH, however, did not change among the groups ( Figures 2C and 2D , respectively).

**Figure 2:** Lung antioxidant measurements. A) Superoxide dismutase activity; B) Catalase activity; C) Glutathione peroxidase activity; D) Total sulfhydryl groups. Data are expressed as mean ± SD. One-way ANOVA with the Student-Newman-Keuls post-hoc test. a P<0.05 vs SHAM; b P<0.05 vs MI. SHAM: Control group; MI: myocardial infarction group; MI + PS: myocardial infarction + pterostilbene .

### Nrf2 protein expression in the lung tissue

Nrf2 expression may be involved with the antioxidant effects of PS. Nrf2 is a protein related with the regulation of antioxidant enzymes transcription and can be stimulated by molecules such as phenolic compounds. Indeed, our data showed that PS treatment promoted a significantly increase in Nrf2 protein expression in the MI+PS group compared to MI group (p<0.05). There was no difference in Nrf2 expression between SHAM and MI groups ( Figure 3 ).

**Figure 3:** Lung’s western blot analysis of Nrf2 expression. A representative gel showing 1 band for each experimental group is provided. Data are expressed as mean ± SD. One-way ANOVA with the Student-Newman-Keuls post-hoc test. b P<0.05 vs MI. SHAM: Control group; MI: myocardial infarction group; MI + PS: myocardial infarction + pterostilbene .

### Evaluation of xanthine oxidase and NADPH oxidase in the RV

Myocardial infarction stimulated pro-oxidant enzymes, which were attenuated by PS administration. In relation to this, xanthine oxidase protein expression was augmented in the RV of MI group in comparison to the other groups (p<0.05). However, in MI+PS group, xanthine oxidase levels were not different from SHAM group, indicating an attenuation of this pro-oxidant enzyme in the infarcted animals treated with PS. In the same way, there was an increase in NADPH oxidase activity in MI group compared to SHAM, which seemed reduced in MI+PS group (p<0.05), showing the contribution of PS treatment to reduce the superoxide anion radical production in RV ( Figure 4A and 4B ).

**Figure 4:** Right ventricle oxidative stress. A) Western blot analysis of xanthine oxidase expression. A representative gel showing 1 band for each experimental group is provided; B) NADPH oxidases activity. Data are expressed as mean ± SD. One-way ANOVA with the Student-Newman-Keuls post-hoc test. a P<0.05 vs SHAM; b P<0.05 vs MI. SHAM: Control group; MI: myocardial infarction group; MI + PS: myocardial infarction + pterostilbene .

### Sulfhydryl concentration and NOS activity in RV

Sulfhydryl concentration (thiol groups relevant as non-enzymatic antioxidants) was decreased in MI group compared to SHAM (p<0.05); however, MI+PS group reestablished the sulfhydryl levels (p<0.05). Besides that, infarcted non-treated rats showed reduced NOS activity when compared to SHAM animals, while treatment with PS recovered this enzyme activity (p<0.05) ( Figure 5A and 5B ).

**Figure 5:** A) Right ventricle total sulfhydryl groups; B) Right ventricle nitric oxide synthase activity. Data are expressed as mean ± SD. One-way ANOVA with the Student-Newman-Keuls post-hoc test. a P<0.05 vs SHAM; b P<0.05 vs MI. SHAM: Control group; MI: myocardial infarction group; MI + PS: myocardial infarction + pterostilbene .

## Discussion

The main finding of this study was to demonstrate that treatment of infarcted rats with PS promoted beneficial effects in the lungs and in the RV. Evaluating the left ventricle morphology and function after infarction, both infarcted groups showed cardiac dilatation, as demonstrated by the increase in cardiac volumes, and contractility impairment, as shown by the decrease in fractional shortening. PS administration, however, attenuated the increase in end-systolic volume, which seems to be a positive result, since the increase in end-systolic volume can be related to the development of lung congestion. In fact, PS treatment prevented lung congestion, evaluated by lung/ body weight ratio. The infarcted perimeter was not different between MI and MI+PS rats, demonstrating the homogeneity of the cardiac injury between these groups. In terms of morphometric parameters, neither the right nor the left ventricle of the infarcted groups presented hypertrophy. These results could be related with the fact that the animals were evaluated only 14 days after infarction. In this time point, a previous study from our group also did not find any difference in these parameters.^32^

The lungs are the organs most affected by heart failure, and pulmonary dysfunction are a key factor for poor clinical outcomes in infarcted patients.^34^ Nevertheless, our study found no changes in total ROS levels in the lungs of infarcted rats. On the other hand, the lungs of MI+PS group showed increased ROS levels, which is in accordance with the reported role of stilbenes in inducing ROS production *in vitro* .^16^ MI group showed an increased in the lipid peroxidation, indicating oxidative damage in the lungs. MI+PS group, however, showed a reduction in lipid peroxidation-induced pulmonary injury, since there was a decrease in TBARS levels. In view of that, a possible hypothesis is that this increase in ROS levels caused by PS administration could represent a hormetic mechanism,^35^ which leads to an increase in the antioxidant defenses, preventing lipid peroxidation. In fact, previous studies with other natural compounds that also present a pro-oxidant effect, such as sulforaphane, have already described this protective mechanism caused by antioxidant system stimulation.^36^ Indeed, PS treatment may provoke an adaptation against increased ROS levels through cellular oxidative changes in SOD and CAT, which are two important enzymes that belong to the first line of defense against oxidative stress.^9^ In our study, reduced SOD activity in the lungs of MI group suggests a deficient protection against anion superoxide radical, which could cause an increase in oxidative stress in later stages of MI.^17^ On the other hand, PS treatment recovered SOD activity in MI+PS group, demonstrating its protective effect in redox homeostasis. Our results showed increased CAT activity in the pulmonary tissue of MI+PS group. Since hydrogen peroxide may react with metals, such as iron, and produce hydroxyl radical,^37^ this increased CAT activity in MI+PS groups arises as an important defense against the production of this radical in the lungs. In terms of non-enzymatic defenses, it was found an increase in GSH concentration in the lungs of MI+PS group. GSH is the most prevalent low-molecular-weight antioxidant peptide^38^ and participates in redox regulation and homeostasis.^39^ In the present study, increased GSH concentration could have contributed to reduced TBARS levels in MI+PS group, decreasing oxidative stress in these animals. Besides enzymatic and non-enzymatic defenses stimulation, PS could also induce improvement in antioxidant profile in the pulmonary tissue through stimulation of cytoprotective proteins, such as Nrf2.^40^

In this context, Nrf2 acts as a redox sensitive transcription factor, playing a key role in pulmonary antioxidant response. In situations of redox balance, Nrf2 is anchored to Kelch-like ECH-associated protein 1 (Keap1). However, when there is a redox homeostasis disruption, Nrf2-Keap1 complex dissociates and release Nrf2, which can translocate into the nucleus, and initiate the transcription of antioxidant molecules.^40^ Indeed, studies in the literature have demonstrated that Nrf2 plays an important role in the synthesis of endogenous antioxidant enzymes.^41^ Moreover, according to Lacerda et al.^15^ PS up-regulates the expression of Nrf2, which increases cellular GSH and mitigates oxidative damage.^15^ In our study, Nrf2 immunocontent levels was increased in the lungs of MI+PS rats, suggesting that PS induces Nrf2 activation, which could be an explanation for the improvement in the antioxidant defenses and the reduction in lipid peroxidation. In view of these data, PS showed to be protective to the lungs after infarction, since prevented pulmonary congestion, increased SOD and CAT activities, increased GSH levels and prevented lipid peroxidation, as well as induced Nrf2, which is a cytoprotective protein.

Regarding the RV, the pro-oxidative scenario of myocardial infarction affects significantly this chamber.^20^ Our results showed that myocardial infarction leads to elevated xanthine oxidase expression in the RV. Wang et al.,^42^ showed increased xanthine oxidase levels in the heart 12 weeks post-infarction, associated with lipid peroxidation and cardiac dysfunction.^42^ Besides that, NADPH oxidase activity, which is an important source of ROS, was also evaluated in our study. This enzyme activity was elevated in MI rats, and PS administration was capable of prevent this increase. The elevated xanthine oxidase levels and NADPH oxidases activities observed predispose the RV to increased superoxide anion concentration, and consequently antioxidant reserve depletion. Corroborating these results, we also found reduced sulfhydryl levels in MI group. MI+PS group, however, showed increased thiol groups content. In the MI group, the elevated superoxide anion concentration, produced by NADPH oxidases and xanthine oxidase, may interfere unfavorably in the ROS/NO balance in the RV. In fact, our results showed reduced NOS activity in the right chamber of MI group. NOS is a relevant enzyme in NO production, which plays critical role in cardioprotection.^43^ In the present study, PS administration prevented NOS activity reduction, which occurred in MI group. Nevertheless, considering the effect of this compound in blunting pro-oxidant enzymes, such as NADPH oxidase and xanthine oxidase, concomitant with partial NOS activity retrieval, PS seems to contribute in the maintenance of a cardioprotective ROS/NO balance in the RV.

## Conclusions

In conclusion, PS administration promoted beneficial effects in the lungs of infarcted animals, decreasing lipid peroxidation and increasing antioxidant defenses, such as SOD and catalase activities and GSH levels. Besides that, this compound prevented the increase in NADPH oxidase activity and in xanthine oxidase expression in the RV of infarcted animals. These results were probably related with an improvement in ROS/NO balance in this chamber. In view of that, our findings suggest that PS effectively presents protective effects in the lungs and RV after myocardial infarction.

**Figure 6:** Graphical abstract .
